# BA-MPCUBIC: Bottleneck-Aware Multipath CUBIC for Multipath-TCP

**DOI:** 10.3390/s21186289

**Published:** 2021-09-19

**Authors:** Imtiaz Mahmud, Tabassum Lubna, Geon-Hwan Kim, You-Ze Cho

**Affiliations:** School of Electronic and Electrical Engineering, Kyungpook National University, Daegu 41566, Korea; imtiaz@knu.ac.kr (I.M.); lubna@knu.ac.kr (T.L.); kgh76@ee.knu.ac.kr (G.-H.K.)

**Keywords:** multipath-TCP, shared bottleneck detection, multipath CUBIC, coupled multipath CUBIC, coupled congestion control

## Abstract

The Congestion Control Algorithm (CCA) in the Multipath Transmission Control Protocol (MPTCP) is fundamental to controlling the flow of data through multiple subflows (SF) simultaneously. The MPTCP CCA has two design goals: first, always ensure better throughput than single path TCP (SPTCP) flows, and second, collectively, MPTCP SFs going through a shared bottleneck (SB) should occupy bandwidth fairly, i.e., close to the bandwidth occupied by an SPTCP flow. Although several MPTCP CCAs exist, they primarily focus on specific scenarios and could not satisfy the design goals in diverse and dynamic scenarios. Recently, CUBIC has become a widely used CCA for SPTCP for its better compatibility with high-speed internet. CUBIC’s effective implementation in the MPTCP is expected to provide improved throughput and fairer behavior, thus satisfying the design goals. However, although the current multipath CUBIC (MPCUBIC) implementation ensures better fairness, it fails to ensure better throughput. We believe the application of same rule for SFs going through an SB and non-shared bottleneck (NSB) makes it difficult for MPCUBIC to adapt to diverse and dynamically changing network scenarios, thus resulting in poor throughput. Therefore, we present an improved version of MPCUBIC, namely bottleneck-aware MPCUBIC (BA-MPCUBIC), to resolve the throughput issue. First, we deploy an innovative bottleneck detection method that successfully differentiates between an SB and NSB based on round-trip-time, enhanced congestion notification, and packet loss. Then, we implement SPTCP CUBIC and MPCUBIC as the CCAs for SFs going through NSBs and SBs, respectively. Extensive emulation experiments demonstrate that the BA-MPCUBIC successfully detects SBs and NSBs with the highest detection accuracy and the lowest detection time compared with other approaches. Moreover, BA-MPCUBIC successfully satisfies the MPTCP design goals in the considered diverse and dynamic scenarios by ensuring both better throughput and fairness.

## 1. Introduction

**Figure 1:**
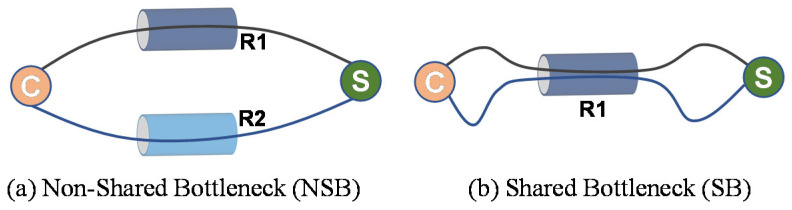
Conceptual representation of—(**a**) non-shared bottleneck (NSB), (**b**) shared bottleneck (SB).

Devices with multiple communication interfaces, such as 5G/4G and WiFi, are widely used. Although it is anticipated that such devices will increase the speed of the Internet by the simultaneous use of multiple interfaces, in practice, intermittently, they are significantly slower than devices using a single communication module [1,2,3]. Lack of a suitable transport layer protocol is one of the key reasons why such devices have not achieved anticipated results. Although the multipath transmission control protocol (MPTCP) has been implemented to utilize multipath communication interfaces simultaneously, the MPTCP has difficulty utilizing the underlying network to its full extent due to the complex network architecture of the Internet [4,5,6,7]. In particular, it is currently unable to properly handle shared bottleneck (SB) and non-shared bottleneck (NSB) links on the Internet, especially in a dynamically changing network environment. In the MPTCP, each flow between the MPTCP server and client is considered a subflow (SF). When two or more SFs travel through a common bottleneck link, that link is considered an SB, otherwise, an NSB. Figure 1 illustrates the concept of SB and NSB.

The congestion control algorithm (CCA) used with the MPTCP plays a vital role in properly utilizing the network. MPTCP CCAs must satisfy two key design goals [8,9,10].

- Goal 1. Improve throughput. MPTCP flows must perform better than single path transmission control protocol (SPTCP) flows.
- Goal 2. Fairness. If two or more SFs go through an SB, collectively their consumed bandwidth (BW) should be similar to the BW consumed by an SPTCP flow going through that SB.

Current MPTCP CCAs have several limitations. For example, popular MPTCP coupled CCAs, such as LIA [9], OLIA [11], BALIA [12], Couple+ [13], D-LIA [14], and D-OLIA [15], behave highly conservatively, i.e., they are aggressively fairer to SPTCP flows; thus, they often fail to utilize the available BW in the NSB [5].

Recently, we proposed C-MPBBR [16], a coupled congestion control algorithm for MPTCP based on Google’s recently proposed BBR CCA [17]. Through extensive emulation experiments, we concluded that C-MPBBR could successfully differentiate between SBs and NSBs, and successfully utilized the underlying network appropriately. However, currently, BBR is still under development and is not widely implemented. Therefore, a stable widely usable MPTCP CCA remains unavailable.

Interestingly, although CUBIC is the most widely used SPTCP CCA on the Internet [18], its MPTCP compatible version is still underdeveloped. To the best of our knowledge, to date, two implementations of CUBIC for MPTCP are available. In 2012, Le et al. proposed an MPTCP CUBIC implementation based on LIA [19]. Their proposed implementation follows the principles of LIA [5,9]; thus, it also inherits the aggressive fairer/conservative behavior of LIA and cannot fulfill the design goals of MPTCP CCAs. In 2020, Kato et al. [20] proposed mpCUBIC for high-speed internet; however, this implementation has a vital shortcoming, i.e., it cannot handle more than two SFs at a time. Moreover, a comprehensive investigation on whether it can fulfill both design goals of MPTCP CCAs has not been undertaken. Therefore, a proper and robust implementation of multipath CUBIC that satisfies the design goals of MPTCP CCAs even in a dynamically changing network environment is still missing.

In this study, we primarily focus on developing an implementation of CUBIC for MPTCP that fulfills both MPTCP CCA design goals and utilizes all SFs effectively even in a dynamically changing network environment. However, to successfully fulfill both the design goals in diverse and dynamic network conditions, we believe that the MPTCP CCA needs to handle SBs and NSBs differently. Thus, it needs to successfully differentiate the SFs going through an SB from those going through an NSB. Although different SB detection techniques are currently available, they have different limitations. For example, the technique proposed by Wei et al. [8] cannot work without explicit congestion notification (ECN). Wei et al. [21] also proposed a technique that only depends on congestion intervals/packet loss intervals. The other SB or NSB detection techniques primarily focus on round-trip-time (RTT) or one-way-delay (OWD) to determine the SB [22,23,24,25]. However, dependence on a single parameter often leads to detection errors and results in poor performance. Thus, there is a considerable scope to improve SB detection techniques to further improve performance. In addition, we believe that a combined and proper utilization of RTT, ECN, and packet loss can significantly improve SB detection results.

Therefore, in this paper, we propose bottleneck-aware multipath CUBIC (BA-MPCUBIC), a CUBIC implementation for MPTCP that effectively enhances the performance of MPTCP in terms of throughput and the fairness index by efficiently utilizing and sharing the underlying network. The primary contributions of this study can be summarized as follows.

1. As a CUBIC-based MPTCP CCA, the proposed BA-MPCUBIC successfully fulfills the design goals of MPTCP CCAs while effectively utilizing all available SFs, even in diverse and dynamically changing network conditions.
2. To differentiate between SFs going through SBs and NSBs, BA-MPCUBIC implements three filters, i.e., RTT_filter, ECN_filter, and PL_filter, based on RTT, ECN, and packet loss, respectively. Two or more SFs are grouped as going through an SB only when any two or all three filters are true for three consecutive acknowledgments (ACK).
3. As SFs going through an SB should be fairer to SPTCP flows, BA-MPCUBIC implements the multipath CUBIC implementation proposed by Le et al. [19] for them.
4. For SFs going through an NSB, the SPTCP CUBIC algorithm is implemented such that the SFs can effectively compete with SPTCP flows and achieve better throughput.
5. BA-MPCUBIC detects SBs and NSBs with a high detection accuracy in a shorter time. It claims its legit share in NSB by ensuring high throughput, while fairly leaving a sufficient share for SPTCP flows in an SB ensuring a high fairness index.
6. We considered seven MPTCP CCAs and experimented with them in five diverse and dynamically changing network scenarios. The results show that BA-MPCUBIC significantly improves the throughput by successfully exploiting all the available paths while ensuring better fairness with SPTCP flows. Among all the considered MPTCP CCAs in all the considered scenarios, BA-MPCUBIC is the best performer in terms of fulfilling the design goals of MPTCP CCAs.

The remainder of this paper is organized as follows. Section 2 summarizes related work. The BA-MPCUBIC algorithm is briefly described in Section 3. The proposed BA-MPCUBIC algorithm is evaluated in comparison to existing CCAs in Section 4. Conclusions and suggestions for future work are presented in Section 5.

## 2. Related Works

In this section, we briefly describe the three well-known MPTCP CCAs as LIA, OLIA, and BALIA, two previously proposed CUBIC-based MPTCP CCAs and some SB detection techniques, their key considerations, and limitations.

Raiciu et al. proposed LIA as a coupled MPTCP CCA to fulfill the design goals of MPTCP CCAs [9]. LIA could successfully utilize all the available paths resulting in better throughput, shift traffic from the more congested path to the less congested path, and ensure fairness with the SPTCP flows. However, later it was reported that LIA shows such an aggressively fairer nature towards SPTCP flows that it even results in less throughput than the SPTCP flows [5].

Khalili et al. reported that LIA leads to a tradeoff between responsiveness and optimal load balancing [11]. They proposed OLIA, an improved version of LIA to ensure the simultaneous existence of both responsiveness and optimal load balancing. However, OLIA also inherits the aggressive fairer nature of LIA and results in poorer throughput than SPTCP flows intermittently [5].

Peng et al. found that, in a changing network condition, OLIA becomes unresponsive to the network changes time to time [12]. They proposed BALIA to resolve this unresponsive nature of OLIA. However, BALIA did not address the aggressive fairer nature of LIA or OLIA. Rather, BALIA also inherits the aggressive fairer nature and results in less throughput that SPTCP flows in times [5].

In an attempt to fulfill both MPTCP CCA design goals and implement CUBIC for MPTCP as a coupled CCA, Le et al. [19] proposed MPCubic following the basic implementation principle of LIA [5,9]. Although they could exploit all paths simultaneously, their implementation shows aggressive fairness toward SPTCP flows, which results in a significantly low throughput in NSBs. This behavior can be attributed to their decision of following the basic principle of LIA.

Recently, Kato et al. [20] proposed mpCUBIC to work as an MPTCP CCA and implemented it in Linux. They also followed the basic principle of LIA [5,9]. Although they claim to achieve better performance even in NSBs, their implementation has a significant limitation, i.e., they can only utilize two SFs at a time. Thus, they cannot satisfy MPTCP’s key goal to exploit all paths simultaneously. Moreover, a proper investigation on whether they satisfy the two MPTCP CCA design goals is absent. Therefore, a proper CUBIC implementation for MPTCP has not yet been realized.

Recently, some researchers have suggested different SB detection techniques for MPTCP. Wei et al. [8] proposed SB-CC that leverages the ECN mechanism to detect SFs going through an SB and the degree of congestion. Then, SB-CC balances the load among all SFs based on the degree of congestion. Although their proposed technique could achieve higher throughput than existing MPTCP CCAs, it does not work in the absence of an ECN mechanism.

Previously, Wei et al. [21] proposed another SB detection mechanism, i.e., SBDV that used the variance of the time interval between congestion events to detect SFs going through an SB. Ferlin et al. [22] proposed an SB detection technique based on skewness, variability, and key frequency of OWD. Similarly, Yousaf et al. [23], Zhang et al. [24], and Kim et al. [25] proposed different SB detection techniques primarily based on either OWD or RTT. However, detecting SB based on a single parameter often leads to false-positive or false-negative detections, and degrades the overall performance both in terms of detection capability and throughput. Therefore, there is a significant scope for research to improve SB detection capability.

Finally, a versatile and widely accepted MPTCP CCA that can satisfy the design goals of MPTCP in diverse and dynamically changing network scenarios is absent till now, to the best of our knowledge. Although CUBIC is well accepted as an SPTCP CCA, its successful variant for MPTCP satisfying the design goal MPTCP CCAs is missing too. This highly motivated us to present this work proposing a multipath CUBIC implementation that can successfully satisfy the design goals of MPTCP CCAs, i.e., ensure high throughput and fairness toward SPTCP flows in diverse and dynamic network scenarios.

## 3. Bottleneck Aware Multipath CUBIC

In this section, we briefly describe the proposed BA-MPCUBIC, the motivation behind it, and the considerations. In addition, we present algorithms for the RTT, ECN, and packet loss filters.

As discussed previously, the proposed BA-MPCUBIC can handle SFs going through both SBs and NSBs efficiently, thereby satisfying the two MPTCP CCA design goals. To accomplish this, we propose using separate methods for SFs traveling through SBs and NSBs. However, we first need to successfully differentiate SFs going through an SB from SFs traveling via an NSB. Therefore, an effective SB and NSB detection technique is required. Because a direct signal from the router to the end hosts to provide the router’s current properties is currently unavailable on the Internet, SB and NSB detection techniques primarily attempt to better estimate SBs and NSBs. However, the currently available SB and NSB detection techniques often return detection errors, primarily because they differentiate based on changes to a single parameter, such as OWD, RTT, ECN, or packet loss. To construct a highly efficient SB and NSB detector, we believe an effective estimation process that intelligently considers all the available information before making the final decision is required. Therefore, we propose to consider RTT, ECN, and packet loss simultaneously. Based on these three parameters, we design three filters, RTT_filter, ECN_filter, and PL_filter. The final decision is made based on the outcome of these three filters.

### 3.1. Considerations and Design of RTT_Filter

In this subsection, we discuss the considerations behind the RTT_filter and present the RTT_filter algorithm.

**Figure 2:**
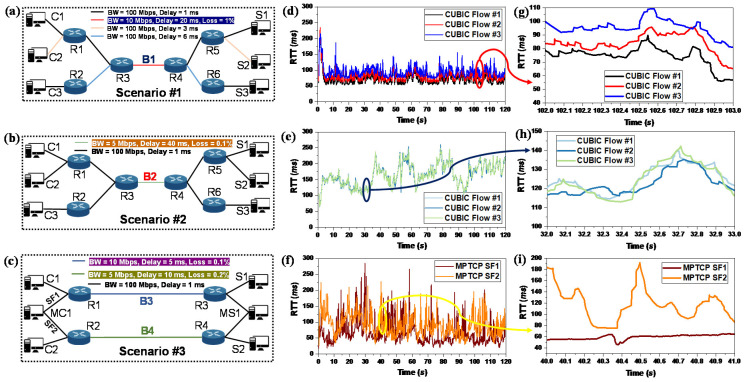
A simple experimental analysis to observe the relationship between RTT and SB; (**a**–**c**) Scenarios #1–3; (**d**–**f**) RTT for Scenarios #1–3; and (**g**–**i**) the magnified version of RTT for Scenarios #1–3 for the period between 102–103 s, 32–33 s, and 40–41 s, respectively.

To understand the considerations for the RTT_filter, we need to understand the relationship between the RTT of different SFs and an SB or NSB. To explain these considerations, we describe a simple experiment. Figure 2a–c show three experimental scenarios, Scenarios #1–3, respectively. In Scenario #1 and #2, there are three SPTCP servers and clients. Here, a single server is connected to only one client; thus, there are three SPTCP flows. Note that, for this experiment, we use CUBIC as the SPTCP CCA. B1 and B2 are the bottleneck links between routers R3 and R4 through which all the three flows travel. The routers have 10 and 5 Mbps BW, delays of 20 and 50 ms, and packet loss of 1% and 0%, respectively. With this configuration, B1 represents a moderate BW link with a small buffer, and B2 represents a highly congested link with a deep buffer. All flows in Scenario #2 have the same RTT, whereas flows in Scenario #1 have different RTTs. The experiment was emulated in Mininet [26]. Figure 2d,e show the measured RTT of all flows for the entire emulation time for Scenarios #1 and #2, respectively. To clearly represent their behavior, Figure 2g,h show a magnified version of the RTTs observed between 102–103 and 30–31 s for Scenarios #1 and #2, respectively. Although the flows in Scenario #1 had different RTTs, and Scenario #2 had an extended delay property, the RTTs show a similar tendency while passing through an SB, i.e., the RTT changes in an almost similar manner for flows passing through an SB.

In contrast, Scenario #3 is designed to observe the RTT changes in an NSB. Here, there is an MPTCP client (MC1) and server (MS1) connected via two different paths (SF1 and SF2), and two SPTCP clients and servers. The SPTCP flows act as the background traffic for the two SFs. The bottlenecks B3 and B4 have distinctive characteristics. The SF1 and SF2 travel via two different bottlenecks B3 and B4, respectively. Thus, B3 and B4 are the NSBs. Note that an uncoupled MPCUBIC was used as the MPTCP CCA for this experiment. For both the SFs, Figure 2f,i show the RTT and magnified RTT between 40–41 s, respectively. As we can observe, the RTT changes are distinctive for the two SFs which indicates that the RTT changes of flows going through different NSBs are different.

**Figure 3:**
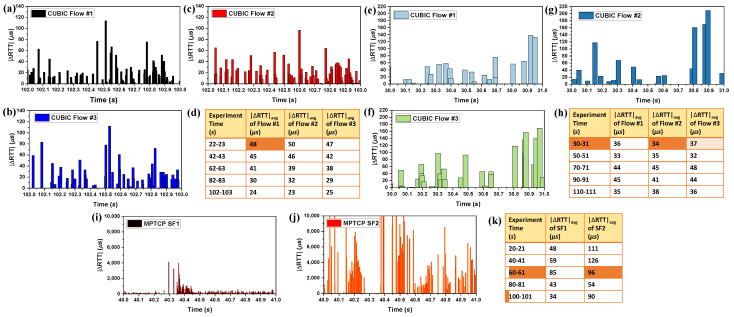
|ΔRTT| and ${\left|\Delta RTT\right|}_{avg}$ for the simple experimental analysis. (**a**–**c**), (**e**–**g**), and (**i**,**j**) show the |ΔRTT| for three CUBIC flows for Scenarios #1 and #2, and two MPTCP SFs for Scenario #3, respectively. (**d**,**h**,**k**) show the ${\left|\Delta RTT\right|}_{avg}$ calculated during different intervals for the three CUBIC flows for Scenario #1 and #2, and two SFs for Scenario #3, respectively.

We also calculated the absolute value of the RTT changes (|ΔRTT|) during each ACK as follows:

(1) $$\left|\Delta RTT\right|=\left|RTT_{i}-RTT_{i-1}\right|$$

where $RTT_{i}$ is the current RTT and $RTT_{i-1}$ is the RTT at the last ACK event. Figure 3a–c and Figure 3e,f show the |ΔRTT| for the three CUBIC flows of Scenarios #1 and #2 during the periods of 102–103 and 30–31 s, respectively. It is evident that they show a similar pattern of RTT changes. Moreover, Figure 3i,j show the |ΔRTT| for the two SFs of Scenario #3 for the period of 40–41 s. It also becomes evident that the SFs show a different pattern of RTT changes.

Furthermore, for each period, we calculated the average |ΔRTT| (${\left|\Delta RTT\right|}_{avg}$) as follows:

(2) $${\left|\Delta RTT\right|}_{avg}=\frac{{\left|\Delta RTT\right|}_{total}}{Q}$$

where ${\left|\Delta RTT\right|}_{total}$ is the total value of |ΔRTT| during the last one second time interval, and *Q* is the number of samples, i.e., the number of |ΔRTT| collected during that time. Figure 3d,h show the values of the calculated ${\left|\Delta RTT\right|}_{avg}$ during different periods for the three flows of Scenarios #1 and #2, respectively. The three flows ${\left|\Delta RTT\right|}_{avg}$ again show the same trend and remain within the ±20% range of each other. Moreover, it is also evident that, for SBs with different properties, ${\left|\Delta RTT\right|}_{avg}$ also differ and generally do not fall in the ±20% range of each other. Furthermore, Figure 3k shows the calculated ${\left|\Delta RTT\right|}_{avg}$ during different periods for the two MPTCP SFs. From Figure 3k, it also becomes evident that ${\left|\Delta RTT\right|}_{avg}$ are different for flows going through different NSBs and do not stay in the range of ±20% with each other in general. Therefore, we can conclude that the SFs going through an SB can be grouped based on ${\left|\Delta RTT\right|}_{avg}$. However, one might argue that the ±20% range may not be true for all possible cases. To satisfy this query we plan to conduct further emulation and real-world experiments and deduct both practical and theoretical analysis in our future work. Note that we do not include them in this work to remove ambiguity and maintain a concise presentation.

Considering the current finding, we design the RTT_filter such that it considers ${\left|\Delta RTT\right|}_{avg}$ as a factor to determine whether an MPTCP SF goes through an SB or an NSB. RTT_filter returns true when it decides that an SF ($SF_{i}$) might be going through an SB, and false otherwise. To design the RTT_filter, we start by deciding the period (RTT_filter_period). For this, we ran several experiments and observed that the longer the RTT_filter_period, the more accurate the measurement. However, increasing the RTT_filter_period has a negative impact on the SB detection time, i.e., the longer the RTT_filter_period, the longer the SB detection time. To balance these two factors, we decided to set the RTT_filter_period as 1 s based on our observations. Thus, the RTT_filter updates the ${\left|\Delta RTT\right|}_{total}$ and *Q* values after the reception of each ACK and calculates the value of ${\left|\Delta RTT\right|}_{avg}$ after an interval of 1 s. To determine whether an $SF_{i}$ is going through an SB or NSB, we consider a set *A* containing all SFs,

(3) $$A=\{SF_{1},SF_{2},SF_{3},.....,SF_{n}\}$$

where *n* is the total number of SFs between the MPTCP client and server. If any other $SF_{j}\in A-\{SF_{i}\}$ has ${\left|\Delta RTT\right|}_{avg}\_SF_{j}$ in the ±20% range of the ${\left|\Delta RTT\right|}_{avg}\_SF_{i}$, we consider that $SF_{i}$ might be going through an SB and return true, otherwise NSB and return false. Algorithm 1 presents a summary of the pseudocode for the RTT_filter.
**Algorithm 1:** BA-MPCUBIC: *RTT_filter***Initialization:**
 *α = 0.2*
 Δ*RTT_total_*
*= 0* Δ*RTT_avg_ = 0*
 *number_of_samples = 0*

 *RTT_max_ = 0*

 *Temp_RTT_max_ = 0*

 *RTT_filter = false*
**Upon reception of ACK:** **if** *system_current_time* < *update_time_of_RTT_filter*
**then**  Δ*RTT_total_* = Δ*RTT_total_* + |*RTT_curr_ − RTT_prev_*|  *number_of_samples* = *number_of_samples* + 1  **if** *Temp_RTT_max_* < *RTT_curr_* **then**   *Temp_RTT_max_* = *RTT_curr_*

  **end if**

 **else**
  *update_time_of_RTT_filter* = *system_current_time* + 1.0 s  Δ*RTT_avg_* = Δ*RTT_total_* ÷ *number_of_samples*  *RTT_filter_max_limit* = Δ*RTT_avg_* + Δ*RTT_avg_* × α  *RTT_filter_min_limit* = Δ*RTT_avg_ −* Δ*RTT_avg_* × α  *RTT_max_* = *Temp_RTT_max_*  *Temp_RTT_max_* = 0  Δ*RTT_total_* = 0  *number_of_samples* = 0
  *RTT_filter = false*
  **for all** subflow *i*
**do**   **if**
*SF_i_* == *SF_curr_* **then**
    **continue**

   **end if**
   **if** Δ*RTT_avg__ SF_i_* > *RTT_filter_min_limit*
**and**
    Δ*RTT_avg__ SF_i_* < *RTT_filter_max_limit* **then**
    *RTT_filter = true*

   **end if**

  **end for**

 **end if**

 **return**
*RTT_filter*


### 3.2. Considerations and Design of ECN_Filter

When designing the ECN_filter, we attempted to take advantage of the existing ECN detection mechanism for SBs and NSBs. Here, we describe the basic ECN mechanism, and then we discuss the design of ECN_filter.

**Figure 4:**
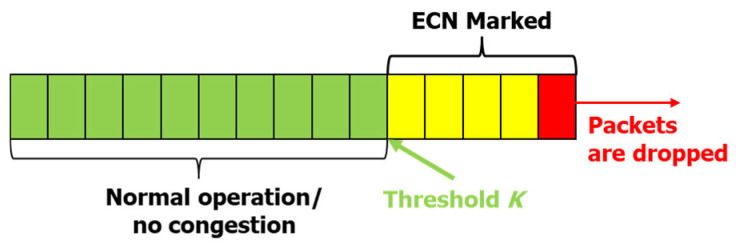
A conceptual sketch of the packet queue of a router detailing the ECN mechanism.

In its simplest form, following [27], ECN-enabled routers have a queue occupancy threshold point *K*. A conceptual diagram that illustrates the basic K threshold function is presented in Figure 4. For an ECN-enabled router, when the average queue length exceeds the *K* threshold, the ECN field for the packets going through that router is set to “11”. When the ECN marked TCP segment is received, the receiver sets the ECN-Echo flag to “1” in the ACK and sends it back to the sender. The sender recognizes the congestion state in an SF from the received ACK.

Based on the number/rate of ECN marked ACKs received, it is possible for the sender to determine a degree of congestion for that specific path [8]. To determine whether an SF is going through an SB or NSB, we consider this degree of congestion. If two or more SFs have a similar degree of congestion, they might be going through an SB, otherwise an NSB. We define the degree of congestion as the rate of reception of the ECN marked ACKs ($ECN_{rate}$) as follows:

(4) $$ECN_{rate}=\frac{ECN\_marked\_ACKs}{received\_ACKs}$$

where received_ACKs and ECN_marked_ACKs are the number of received ACKs and received ECN marked ACKs during the last ECN_filter_period, respectively. We set the ECN_filter_period value to 0.25 s. In summary, we consider that an $SF_{i}$ might be going through an SB only when any other $SF_{j}\in A-\{SF_{i}\}$ shows $ECN_{rate}\_SF_{j}$ in the ±20% range of the $ECN_{rate}\_SF_{i}$. In this case, the ECN_filter returns true, false otherwise. The ECN_filter algorithm is summarized in Algorithm 2. Note that we set the ±20% range and ECN_filter_period value of 0.25 s based on the finding from our extensive and diverse experiments. Moreover, considering the concise presentation of this work, we plan to present the findings of different ranges and different ECN_filter_period values in our future work.
**Algorithm 2:** BA-MPCUBIC: *ECN_filter***Initialization:** *β* = 0.2 *received_ACKs* = 1 *ECN_marked_ACKs* = 1 *ECN_filter* = *false***Upon reception of ACK:** **if**
*system_current_time* < *update_time_of_ ECN_filter* **then**  *received_ACKs = received_ACKs +* 1  **if**
*ECN_marked_ACK* = true **then**
   *ECN_marked_ACKs = ECN_marked_ACKs + 1*

  **end if**

 **else**
  *ECN_rate_* = *ECN_marked_ACKs* ÷ *received_ACKs*  *update_time_of_ ECN_filter* = *system_current_time* + 1.0 sec  *ECN_filter_max_limit* = *ECN_rate_* + *ECN_rate_* × *β*  *ECN_filter_min_limit* = *ECN_rate_ − ECN_rate_* × *β*  *received_ACKs =* 1  *ECN_marked_ACKs* = 1  *ECN_filter* = *false*  **for all** subflow *i*
**do**   **if**
*SF_i_* == *SF_curr_*
**then**
    **continue**

   **end if**
   **if**
*ECN_rate__SF_i_* > *ECN_filter_min_limit*
**and**
*ECN_rate__SF_i_* < *ECN_filter_max_limit*
   **then**
    *ECN_filter* = *true*
   **end if**

  **end for**

 **end if**

 **return**
*ECN_filter*


### 3.3. Considerations and Design of PL_Filter

Packet loss is known to indicate congestion [28,29]. Other factors, such as routing failures, link disruptions, or bit errors, can also cause packet loss; however, these factors occur randomly, i.e., typically they do not occur in all flows going through a bottleneck simultaneously. When the bottleneck queue becomes full, all the packets received afterward are dropped. Thus, this causes packet loss to occur in all flows going through that bottleneck, i.e., packet losses are experienced by all flows simultaneously or at a close interval. We propose to design the PL_filter based on this phenomenon.

We define a period (PL_filter_period) in which, if two or more SFs experience packet loss, we consider that they might be going through an SB. PL_filter_period is defined as follows:

(5) $$PL\_filter\_period\_SF_{i}=3\times RTT_{\max}\_SF_{i}$$

where $RTT_{\max}\_SF_{i}$ is the maximum RTT for $SF_{i}$ observed during the last RTT_filter_period. $RTT_{\max}\_SF_{i}$ is updated after each 1 s interval following an RTT_filter_period. The update algorithm for the $RTT_{\max}$ is shown in Algorithm 1. The PL_filter_period has been set to three times the $RTT_{\max}$ so that we can follow the packet drops for the last three packets for an SF. Now, we consider that an $SF_{i}$ might be going through an SB only when any other $SF_{j}\in A-\{SF_{i}\}$ has experienced a packet loss event during the PL_filter_period and set the PL_filter value to true, and false otherwise. The PL_filter algorithm is summarized in Algorithm 3.
**Algorithm 3:** BA-MPCUBIC: *PL_filter***Initialization:** *last_packet_loss_time* = 0 *PL_filter* = 0**Upon reception of ACK:** *PL_filter* = *false* *last_packet_loss_time* = *system_current_time* *PL_filter_period* = *system_current_time* − (3 × *RTT_max_*) **for all** subflow *i*
**do**  **if**
*last_packet_loss_time_of_SF_i_* > *PL_filter_period*
**then**   *PL_filter* = *true*
  **end if**

 **end for**

 **return**
*PL_filter*


### 3.4. Synchronization between the RTT_Filter, ECN_Filter, and PL_Filter

For the three filters to function properly, they need to be time-synchronized across different SFs of an MPTCP client/server. Therefore, we implement a time synchronization mechanism for the SFs. Note that, as all SFs are running on the same machine, the system time is the same. Therefore, synchronizing the update times of the RTT_filter and PL_filter would be sufficient. To achieve that, whenever an SF starts, it synchronizes its RTT_filter and PL_filter update times with the existing SFs RTT_filter and PL_filter update times. However, the SF that starts first sets the values independently. Algorithm 4 summarizes the mechanism.
**Algorithm 4:** BA-MPCUBIC: Time synchronization between the SFs for *RTT_filter, ECN_filter, and PL_filter***Initialization:**
 *current_time = system_current_time*
 *update_time_of_RTT_filter* = *current_time* + 1.0 sec *sync* = *false***Upon reception of ACK:** *temp_update_time* = *system_current_time +* 3.0 sec **if**
*sync* = *false*
**then**
  **for all** subflow *i*
**do**   **if**
*current_time* < *update_time_of_ RTT_filter_ SF_i_*
**and**
*temp_update_time* > *update_time_of_ RTT_filter_ SF_i_*
**then**    *temp_update_time* = *update_time_of_ RTT_filter_ SF_i_*

   **end if**
   *update_time_of_ RTT_filter* = *temp_update_time*   *update_time_of_ ECN_filter* = *temp_update_time*   *sync* = *true*
  **end for**

 **end if**


### 3.5. Decision on Whether an SF Is Going through an SB or NSB

To this point, we have explained the design process of the three proposed filters. Each filter returns a Boolean true or false decision, implying that an $SF_{i}$ might be going through an SB or NSB, respectively. Now, to finalize the decision on whether an $SF_{i}$ is going through an SB or NSB, we follow the subsequent Boolean expression:

(6) $$\begin{array}{l} (RTT\_filter\;\mathrm{AND}\;ECN\_filter)\;\mathrm{OR}\;(ECN\_filter\;\\ \mathrm{AND}\;PL\_filter)\;\mathrm{OR}\;(PL\_filter\;\mathrm{AND}\;RTT\_filter) \end{array}$$

**Figure 5:**
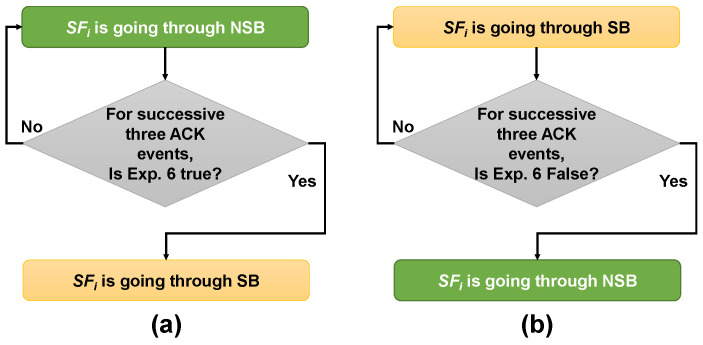
Flowchart on the decision process on whether an $SF_{i}$ is going through an SB or an NSB; (**a**) considering an $SF_{i}$ is currently going through an NSB, and (**b**) considering an $SF_{i}$ is currently going through an SB.

Considering a dynamic network scenario, first, we consider that an $SF_{i}$ is currently going through an NSB. Here, we assume that an $SF_{i}$ is going through an SB only if Equation (6) is true for three consecutive ACK events, and NSB otherwise. In other words, for three successive ACK events, if at least any of the two filters are true for an $SF_{i}$, that $SF_{i}$ is going through an SB, if not then that $SF_{i}$ is going through an NSB. We consider waiting for three successive ACK events to avoid false-positive detections. The flowchart shown in Figure 5a summarizes the decision mechanism.

Now, suppose that an $SF_{i}$ is currently going through an SB. Here, we again apply Equation (6) and observe whether Equation (6) is false for three successive ACK. If false, then that $SF_{i}$ is going through an NSB, and otherwise an SB. Again, we wait for three consecutive ACK events to avoid false-negative detections. The flowchart in Figure 5b summarizes this method.

### 3.6. Applying Different CCAs for SFs Going through SBs and NSBs

To this point, the proposed BA-MPCUBIC successfully isolates the SFs going through the SB from SFs traveling via an NSB. To fulfill the MPTCP CCA design goals, we implement the SPTCP CUBIC CCA for the flows traveling via an NSB such that the NSB SFs can well-compete with the SPTCP flows going through that NSB. For an $SF_{i}$ going through an NSB, the window growth function $W{\left(t\right)}_{i}$ is defined by the following function [18]:

(7) $$W{\left(t\right)}_{i}=C{\left(t_{i}-K_{i}\right)}^{3}+W_{\max,i}$$

where *C* is a CUBIC function, $t_{i}$, $K_{i}$, and $W_{\max,i}$ are the time elapsed from the last packet loss event for $SF_{i}$, the period required for the window growth function to reach $W_{\max,i}$ for $SF_{i}$, and the congestion window (CWND) size prior to CWND reduction during the packet loss event for $SF_{i}$, respectively. $K_{i}$ is calculated as follows:

(8) $$K_{i}=\sqrt[{3}]{\frac{\beta W_{\max,i}}{C}}$$

where β is the multiplicative factor for window reduction during packet loss events.

For the SFs going through the SB, we implement the multipath CUBIC CCA proposed by Le et al. [19] so that the MPTCP SFs behave more fairly with the SPTCP flows going through that SB. The CWND growth function for a $SF_{i}$ going through an SB is defined as follows.

(9) $$W{\left(t\right)}_{i}=\min\left(\delta C{\left(t_{i}-K_{i}\right)}^{3},C{\left(t_{i}-K_{i}\right)}^{3}\right)+W_{\max,i}$$

here, δ denotes the aggressiveness level of the CWND growth function so that MPCUBIC SFs sharing an SB can be fair to SPTCP flows. For flows going through an SB, $K_{i}$ is calculated as follows.

(10) $$K_{i}=\sqrt[{3}]{\frac{\beta W_{\max,i}}{\min(\delta ,1)C}}$$

Interested readers are encouraged to refer to previous studies [18,19] for further details.

### 3.7. Implementation in the Linux Kernel

For implementing the BA-MPCUBIC in the Linux Kernel, we mainly modified tcp_cubic.c file so that it supports and successfully handles MPTCP. Upon reception of each ACK and 3-dup ACKs, cubictcp_cong_avoid and cubictcp_recalc_ssthresh functions are called, respectively. We implemented the key logic for choosing between SPCUBIC CCA and multipath CUBIC CCA in these functions. We implemented separate functions for both the CCAs and the three filters; and updated their internal parameters each time cubictcp_cong_avoid and cubictcp_recalc_ssthresh functions are called. Moreover, we modified the tcp_probe.c module file to continuously observe the internal parameters for debugging.

We found smooth transitions between the two CCAs as we updated the internal parameters of both the CCAs continuously. Moreover, waiting for three successive ACKs for making the decision on transition between CCAs reduces the sudden transitions between the CCAs.

## 4. Performance Evaluation

In this section, we evaluate the performance of BA-MPCUBIC in a wide range of scenarios that are specifically designed to observe how BA-MPCUBIC fulfills the design goals of MPTCP CCAs. We compare the performance of BA-MPCUBIC with conventional MPTCP CCAs, such as LIA, OLIA, and BALIA. In addition, to compare its performance with an uncoupled MPCUBIC implementation, we implemented uncoupled multipath CUBIC (U-MPCUBIC), a variant of CUBIC designed for MPTCP where each flow follows an SPTCP implementation of CUBIC. We also implemented Le’s multipath CUBIC (Le’s MPCUBIC) as a coupled multipath CUBIC implementation. In addition, to compare SB and NSB detection performance and to grasp its impact on our proposed approach, i.e., to use SPTCP CUBIC CCA for SFs going through an SB and Le’s MPCUBIC for SFs going through an NSB, we implemented a modified version of the SB detection algorithm proposed by Ferlin et al. [22], which we refer to as “Ferlin’s SBD+MPCUBIC”. Here, we followed the SB/NSB detection result found by their algorithm and implemented the MPTCP CCA based on process described in Section 3.6 F.

Note that, for each test case, we executed at least 30 experiments. The experimental time was 120 s unless specified otherwise. The results shown in Figures 7 and 8 include the mean, standard deviation, median, 25% and 75% percentiles, and the degree of dispersion.

### 4.1. Experimental Setup

We conducted the performance evaluation and comparison via emulation experiments on a Linux network namespace-based Mininet emulator [26]. We enabled “fq” [30] as the queueing discipline; “ethtool” [31] and “NetEm” [32] were used to configure BW and RTT, respectively; “iperf3” [33] was utilized to transmit the data between a server and a client and to measure total throughput; “ifstat” [34] was used to measure throughput per flows; and “tcpprobe” [35] was employed to measure the CWND, and other internal parameters were used to measure the SB or NSB detection time and accuracy. MPTCP v0.93.4 was deployed and the Linux kernel v4.9.169 was used to conduct the experiments.

### 4.2. Considered Scenarios for Performance Evaluation

**Figure 6:**
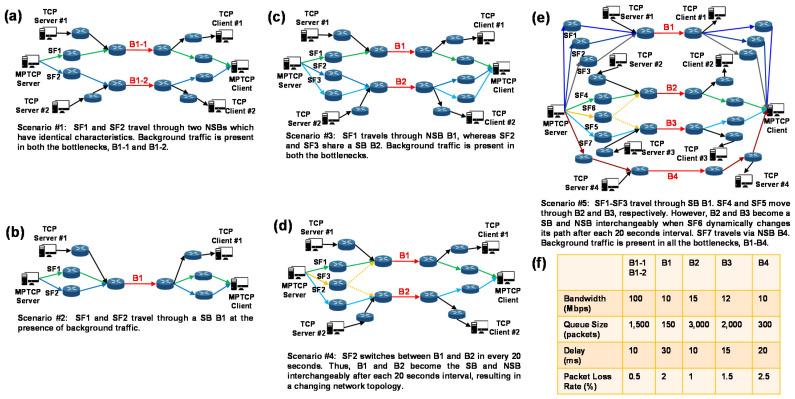
Schematic of the considered scenarios for the performance evaluation. (**a**–**e**) Scenarios #1–5, (**f**) properties of different bottleneck links contemplated in the considered scenarios.

Our objective was to observe how the MPTCP CCAs performed with respect to the design goals, i.e., improve throughput and ensure fairness, and considering the real-world complex Internet. To this end, we designed several scenarios, as shown in Figure 6. In all the scenarios, an MPTCP client and an MPTCP server are connected through a different number of SFs. Note that, in the remaining text, an SPTCP flow with CUBIC as the CCA is considered the background traffic unless stated otherwise.

In Scenario #1, there are two SFs, each of which follows separate paths; thus, B1-1 and B1-2 are the NSBs. Background traffic is present in both paths. This scenario is designed to observe how well the considered CCAs utilize the underlying network.

In Scenario #2, there are two SFs that travel through SB B1, in the presence of background traffic. This scenario is designed to observe how fair the considered CCAs behave with SPTCP flows.

In Scenario #3, there are three SFs. SF1 goes through NSB B1, and SF2 and SF3 travel via SB B2. Background traffic is present in both B1 and B2. Thus, this scenario presents two challenges to the MPTCP CCAs, i.e., to grasp a fair share of BW for SF1 while competing with SPTCP in the NSB B1 and to ensure a fair share of BW for the SPTCP flows in SB B2.

Scenario #4 presents a more interesting situation where SF1 and SF2 travel via B1 and B2, respectively. Background traffic is present in both paths. However, the SF3 path changes after each 20 s interval, i.e., for the first 20 s, SF3 travels via B1, in the next 20 s SF3 goes through B2, in the next 20 s SF3 travels via B1, and so on. As a result, for the first 20 s, B1 is the SB, in the next 20 s B2 is the SB, and in the next 20 s, B1 is the SB. Therefore, B1 and B2 become the SB interchangeably after each 20 s interval. The MPTCP CCAs need to realize this dynamic environment in the network, categorize the flows accordingly, i.e., determine which are going through an SB and which are going through an NSB, and allocate the proper CWND for each SFs. This also challenges the efficacy of the SB or NSB detection mechanism.

Finally, Scenario #5 is a mixture of Scenarios 1-4. Thus, the MPTCP CCAs face all of the above-mentioned challenges simultaneously. SFs1–3 travel via SB B1 in the presence of background traffic. These SFs need to be fair with the SPTCP flow. SF7 travels via NSB B4 in the presence of background traffic. NSB B4 should attempt to attain a similar BW as an SPTCP flow. SFs4–5 travel via B2 and B3, respectively, and, following Scenario #4, SF6 interchangeably uses B2 and B3 after each 20 s interval. Consequently, B2 and B3 become the SB and NSB successively after each 20 s interval. This unique network scenario challenges the MPTCP CCAs in almost all possible ways, gives the experiment a near real-world flavor in a controlled way, and provides the readers with a better understanding of the BA-MPCUBIC and considered MPTCP CCAs performance.

### 4.3. Performance Evaluation in Terms of Aggregate Benefit and Jain’s Fairness Index

As MPTCP CCAs govern multiple SFs simultaneously, measuring only the throughput does not clearly reflect the proper network utilization. Paasch et al. defined a parameter “Aggregate Benefit” (Agre_Bft) to better capture the network utilization by the MPTCP SFs [36]. They considered the goodput and available resources (i.e., BW) of the MPTCP SFs as follows:

(11) $$Agre\_bft=\left\{ \begin{array}{l} \frac{\Psi -\Phi_{\max}}{\sum_{p=1}^{q}\Phi_{p}-\Phi_{\max}}\;,\;\mathrm{if}\;\Psi \geq \Phi_{\max}\\ \frac{\Psi -\Phi_{\max}}{\Phi_{\max}}\;,\;\mathrm{if}\;\Psi <\Phi_{\max} \end{array}\right.$$

where Ψ, $\Phi_{\max}$, $\Phi_{p}$, and *q* are the total goodput acquired by the MPTCP SFs, the maximum available BW among all SFs, the actual available BW for $SF_{p}$ going through path *p*, and the total number of SFs, respectively. The Agre_Bft value ranges from −1 to 1 where a larger value indicates better network utilization. Moreover, Agre_Bft>0 indicates that the usage of MPTCP yields a benefit over SPTCP, and there is no benefit over SPTCP otherwise.

To better understand how fair the considered MPTCP CCAs behave with the SPTCP background traffic, for the MPTCP SFs and SPTCP flows going through a link, we obtain the Jain’s fairness index defined as follows [37,38]:

(12) $$\mathrm{Fairness}\;\mathrm{Index}=\frac{{\left|\sum_{x=1}^{z}\sigma_{x}\right|}^{2}}{z\sum_{x=1}^{z}\sigma_{x}^{2}}$$

where $\sigma_{x}$ and *z* are the allocated BW from the total BW of a link to a flow *x* and the total number of flows going through the link, respectively. Fairness index values range from 0 to 1; the closer the value is to 1, the fairer the CCA.

**Figure 7:**
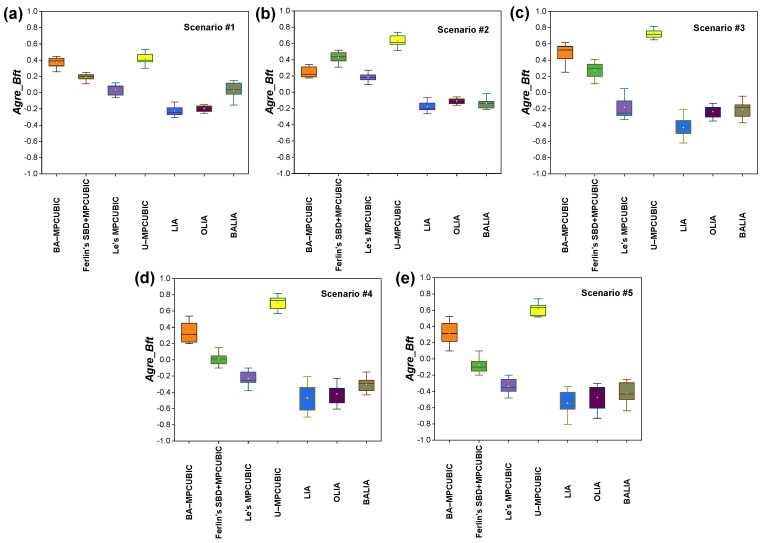
Performance comparison in terms of Agre_Bft for the considered MPTCP CCAs. (**a**–**e**) Agre_Bft for Scenarios #1–5.

**Figure 8:**
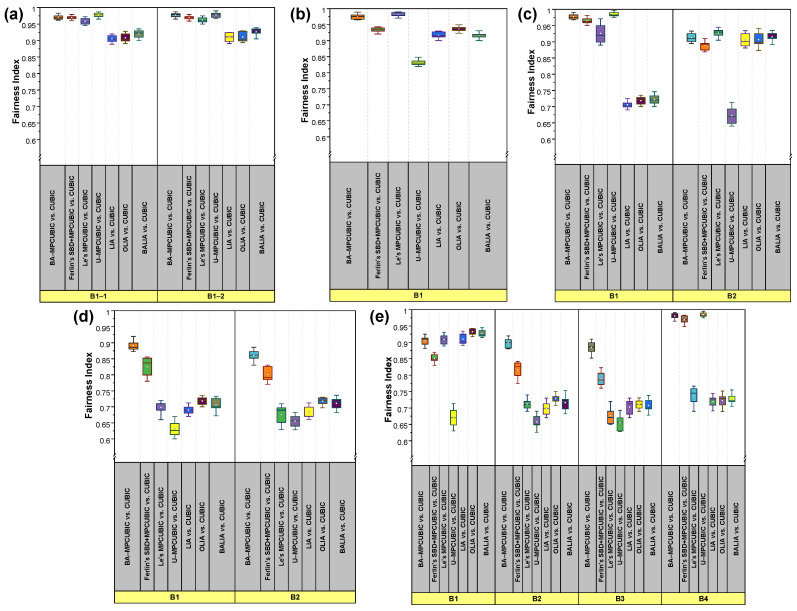
Performance comparison in terms of fairness index for the considered MPTCP CCAs. (**a**–**e**) fairness index for Scenarios #1–5.

Figure 7a and Figure 8a show the Agre_Bft and fairness index for the considered MPTCP CCAs in Scenario #1. Here, the two MPTCP SFs travel through two separate paths via NSB B1-1 and B1-2. Background traffic is present in both B1-1 and B1-2. SF1 and SF2 should behave as two separate SPTCP flows to obtain an equal share of BW in the presence of the background traffic. We can observe that BA-MPCUBIC and U-MPCUBIC attain the highest and almost equal Agre_Bft. As previously described, U-MPCUBIC has been implemented as an MPCUBIC CCA where each SF follows the design of an SPTCP CUBIC CCA. As BA-MPCUBIC realizes an equal Agre_Bft as U-MPCUBIC, BA-MPCUBIC succeeds in its goal to behave as an SPTCP CCA while going through the NSBs. Interestingly, BA-MPCUBIC better utilizes the network compared with Ferlin’s SBD+MPTCUBIC. This is because Ferlin’s SBD+MPCUBIC encounters false-positive decisions from time to time and considers that the two SFs are going through an SB. In such cases, it reduces the CWND and fails to hold an equal share of BW at all times. We believe that the detection errors occur because Ferlin et al. [22] base their decision only on the OWD. Moreover, Le’s MPCUBIC achieves smaller Agre_Bft than BA-MPCUBIC because it follows the same LIA design principle and attempts to behave very fairly with SPTCP flows even in the NSBs. However, Le’s MPCUBIC obtains better Agre_Bft values than LIA, OLIA, and BALIA because it implements CUBIC as a CCA rather than TCP NewReno [39]. Due to the aggressive fairer nature, LIA, OLIA, and BALIA show poorer performance than the others, as was reported in a previous study [5]. Considering the fairness issue, because one MPTCP SF and one SPTCP flow travel in both B1-1 and B1-2, the total BW should be divided in a 1:1 ratio. Thus, an MPTCP SF and an SPTCP flow should each occupy 5 Mbps of BW in both B1-1 and B1-2, respectively. From Figure 8a, it is clearly evident that, compared to other approaches, both BA-MPCUBIC and U-MPCUBIC achieve the highest fairness index because they implement the SPTCP CUBIC CCA in NSBs. Moreover, the better NSB or SB detectability helped BA-MPCUBIC realize a higher fairness index than Ferlin’s SBD+MPCUBIC. The reason given previously for the better Agre_Bft value also applies to the better fairness index compared with Le’s MPCUBIC, LIA, OLIA, and BALIA.

For the considered MPTCP CCAs, Figure 7b and Figure 8b demonstrate the Agre_Bft and fairness index for Scenario #2. Here, both SF1 and SF2 travel through an SB B1 with background traffic. Because the two SFs share a common bottleneck with an SPTCP flow, together they should occupy the BW occupied by an SPTCP flow. In other words, the total throughput of the MPTCP SFs should be equal or close to that of the SPTCP flow. In this scenario, Le’s MPCUBIC performs the best among all considered CCAs. It showed the best fairness index and achieved a decent Agre_Bft value. Because Le’s MPCUBIC is based on LIA and CUBIC, it could ensure better fairness and realize a fair share of BW while competing with CUBIC as the SPTCP CCA. BA-MPCUBIC’s performance was very close to that of Le’s MPCUBIC with a slightly decreased fairness index and increased Agre_Bft value. BA-MPCUBIC initially takes some time to recognize that the SFs are going through an SB. This initial recognition time is the key that defines this difference. Nevertheless, BA-MPCUBIC shows a highly efficient performance compared to its competitors. In contrast, we can observe that U-MPCUBIC realized the highest Agre_Bft value but results in the lowest fairness index. This is because it continues sending data following SPTCP CCA behavior even in the SBs. Ferlin’s SBD+MPCUBIC also shows a similar trend as U-MPCUBIC with a comparatively lower Agre_Bft value and a better fairness index than U-MPCUBIC. As explained earlier, Ferlin’s SBD+MPCUBIC frequently encounters false-positive and false-negative detections. Although the flows go through the SBs, it sometimes considers that they are going through an NSB. Thus, Ferlin’s SBD+MPCUBIC unfairly receives greater BW, which results in a lower fairness index. LIA, OLIA, and BALIA show a considerably better fairness index in this scenario; however, they fail to obtain sufficient BW. We believe this is because LIA, OLIA, and BALIA are based on TCP NewReno, while the SPTCP flow uses CUBIC as the CCA.

For Scenario #3, the performance comparison of the considered CCAs in terms of Agre_Bft and fairness index are shown in Figure 7c and Figure 8c, respectively. Note that Figure 8c shows the fairness index for each of the bottleneck links. Scenario #3 is a mixed scenario that includes both MPTCP CCA design goal challenges. SF1 goes through NSB B1 and SF2 and SF3 travels via the same SB B2. Background traffic is present in both B1 and B2. Therefore, SF1 should behave like an SPTCP flow to obtain an equal share of BW while sharing B1 with the background traffic. SF2 and SF3 should behave fairly following Scenario #2. In this mixed scenario, BA-MPCUBIC demonstrates the best results, i.e., it achieves a high fairness index in both the bottlenecks and ensures high BW utilization thanks to the state-of-the-art bottleneck detection and response algorithm. Although U-MPCUBIC achieves the highest Agre_Bft value, it behaves highly unfairly in B2 following Scenario #2. The unconditional implementation of SPTCP CUBIC CCA as the MPTCP CCA is the key reason behind this behavior. Ferlin’s SBD+MPCUBIC achieves a lower Agre_Bft value and fairness index in both the bottlenecks compared with BA-MPCUBIC. The reason explained previously also applies here. Le’s MPCUBIC ensures better fairness in B2 but fails to maintain this fairness in B1. In addition, the Agre_Bft value is significantly low compared with BA-MPCUBIC, Ferlin’s SBD+MPCUBIC, and U-MPCUBIC. For the aggressive fairer tendency inherited from LIA, Le’s MPCUBIC handles the situation in B2 efficiently but fails to do so in B1. Comparably to the previous scenarios, LIA, OLIA, and BALIA results showed similar tendencies. However, following Le’s MPCUBIC, they achieve better fairness in B2 but fail to maintain it in B1. They also return significantly lower Agre_Bft values. We believe the TCP NewReno based behavior, which competes with SPTCP CUBIC as the background traffic, is the reason for their worse performance.

Scenario #4 introduces an exclusive task to cope with dynamically changing network topology. Here, the SF3 changes its path after each 20 s interval resulting in changes in SB and NSB in real-time. Whenever SF3 goes through B1, B1 becomes the SB as SF1 and SF3 travel through it together, and B2 becomes the NSB. In both B1 and B2, an SPTCP CUBIC flow is present as background traffic. Thus, SF1 and SF3 together should behave like an SPTCP flow to be fair with the background traffic, whereas SF2 should behave like an SPTCP flow so that it can take a proper share of BW while competing with the background traffic. Figure 7d and Figure 8d show the performance comparison results of the considered CCAs in terms of Agre_Bft values and fairness index. We show the fairness index for each of the bottleneck links. In this changing network scenario, BA-MPCUBIC again proves to be the best performer compared with others, i.e., BA-MPCUBIC achieves the best fairness index and ensures better BW utilization. We believe the advanced SB and NSB detection mechanism is the key to this high performance. U-MPCUBIC again achieves the best Agre_Bft values; however, it results in the lowest fairness index because SPTCP CUBIC is implemented as the CCA without any awareness about the bottleneck condition. Ferlin’s SBD+MPCUBIC showed the second-best performance. The reason given for the performance in Scenario #2 also applies here. Le’s MPCUBIC neither obtains a better fairness index nor Agre_Bft values in this scenario. We believe that the absence of an SB or NSB detection mechanism renders Le’s MPCUBIC ineffective in this scenario. The inherited aggressive fairer nature dominates here and causes the three MPTCP flows collectively to behave like an SPTCP flow. The same phenomenon is also true for LIA, OLIA, and BALIA. Thus, they also yield much poorer Agre_Bft and fairness index values in this scenario.

All the challenges demonstrated in Scenarios #1–4 are present simultaneously in Scenario #5. There are seven SFs between the MPTCP client and server. SF1–3 travel through SB B1. SF4 and SF5 travel through B2 and B3, respectively. SF6 changes its path between B2 and B3 after each 20 s interval. Thus, B2 and B3 interchangeably become an SB and NSB after each 20 s interval. SF7 travels via NSB B4. Background traffic is present in all four bottleneck links. Collectively, the three SFs going through SB B1 should take BW close to that taken by an SPTCP flow. SF7, which competes with the SPTCP flow in B4, should try to behave similar to an SPTCP flow to grasp an equal share. When B2 becomes the SB, together SF4 and SF6 should behave like an SPTCP flow, whereas when B3 becomes the SB, SF5 and SF6 should together behave like an SPTCP flow. Figure 7e and Figure 8e show the Agre_Bft and fairness index values for the considered CCAs in Scenario #5, respectively. Again, we show the fairness index for each bottleneck link in Figure 8e. BA-MPCUBIC performs the best in this complex network scenario considering the two MPTCP CCA design goals. BA-MPCUBIC achieves a comparatively better fairness index in all four bottleneck links. At the same time, it could attain a fair and comparatively better BW share, again thanks to its state-of-the-art bottleneck detection and response algorithm. As observed in the previous scenarios, U-MPCUBIC attains the maximum Agre_Bft; however, it returns the minimum fairness index in all bottleneck links except B4 because it adopts the SPTCP CCA mechanism irrespective of bottleneck conditions. In B4, U-MPCUBIC has the highest fairness index because there is only one SPTCP CUBIC flow as the background traffic, and SF7 acts as the SPTCP CUBIC flow by implementing U-MPCUBIC. This result verifies that, although U-MPCUBIC fulfills Goal 1 of MPTCP CCAs, it completely fails to fulfill Goal 2. Similar to Scenario #4, Ferlin’s SBD+MPCUBIC secures comparatively better Agre_Bft and fairness index values in all bottlenecks; however, the results are significantly less than those of BA-MPCUBIC. As explained previously, Ferlin’s SBD+MPCUBIC implements the same reaction algorithm as BA-MPCUBIC when SBs or NSBs are detected. The difference between Ferlin’s SBD+MPCUBIC and BA-MPCUBIC is the SB and NSB detection mechanism. Therefore, we believe that the decrease in performance by Ferlin’s SBD+MPCUBIC compared with BA-MPCUBIC is due to the detection of false-positive or negative SBs or NSBs. We further believe that its sole dependency on a single parameter (i.e., OWD) for the decision-making process is the key reason behind this degraded performance. As was observed in Scenario #4, BA-MPCUBIC significantly outperforms Le’s MPCUBIC, LIA, OLIA, and BALIA in terms of both Agre_Bft and fairness index. The reasons described previously also explain this poor performance. Therefore, it is evident that BA-MPCUBIC performs the best considering the fulfillment of the two MPTCP CCA design goals.

Finally, Table 1 presents the overall performance comparison of the considered MPTCP CCAs in Scenarios #1–5 in terms of Agre_Bft and fairness index. Note that here we represent the average Agre_Bft. For the fairness index, we calculate an average fairness index considering the fairness indexes observed in all the bottlenecks in a scenario. From Table 1, only BA-MPCUBIC and U-MPCUBIC could always ensure an Agre_Bft>0 indicating that they could always ensure an incentive for using MPTCP over SPTCP. Although U-MPCUBIC yields a better Agre_Bft, it results in a very poor fairness index in most of the scenarios in comparison with BA-MPCUBIC. The previously mentioned reasons are valid for the comparatively poor performance of the other considered MPTCP CCAs. Therefore, it becomes evident that BA-MPCUBIC is the best performer in terms of Agre_Bft and fairness index with SPTCP flows, thus best satisfies the MPTCP CCAs’ design goals among the considered MPTCP CCAs in the experimented scenarios.

### 4.4. Performance Evaluation in Terms of Bottleneck Detection Time and Accuracy

In the previous section, we demonstrated and compared the performance of BA-MPCUBIC relative to fulfilling the MPTCP CCA design goals. In this section, we focus on how the BA-MPCUBIC algorithm performs in detecting SBs and NSBs. Initially, we consider how the proposed algorithm responds to changing network scenarios. Then, we compare the bottleneck detection time and detection accuracy for the considered scenarios.

**Figure 9:**
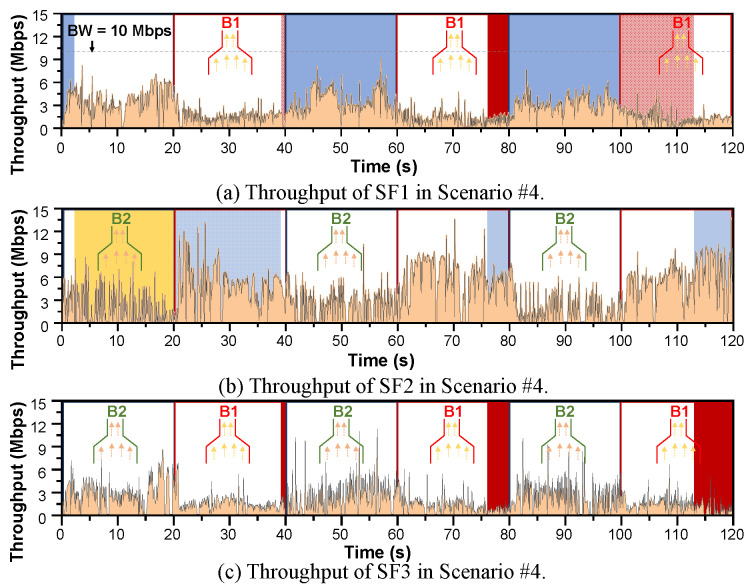
Throughput of the three MPTCP SFs in a randomly selected experiment in Scenario #4; (**a**–**c**) throughput for SF1–3, respectively.

We designed Scenario #4 such that the SB and NSB change every 20 s. Therefore, we selected Scenario #4 to observe the BA-MPCUBIC’s response to a changing network scenario. We randomly selected an experiment in Scenario #4 and plotted the throughput curve of the three SFs, as shown in Figure 9. In this experiment, B1 and B2 become NSB and SB, respectively for the first 0–20 s, SB and NSB for the 20–40 s period, NSB and SB for the 40–60 s period, and so on. We observe that whenever the bottleneck changes, BA-MPCUBIC quickly recognizes the changes and applies the relevant algorithm. Even the SFs going through the SB share the available BW equally. For example, consider a 20–40 s interval when B1 is the bottleneck link and SF1 and SF3 travel via SB B1. B1 has a BW capacity of 10 Mbps. As SPTCP CUBIC flow is present as background traffic, the total available BW for both the SFs becomes 5 Mbps. SF1 and SF3 share this BW evenly. On the other hand, B2 has a BW capacity of 16 Mbps. As B2 is an NSB in the 20–40 s interval, SF2 gets an available capacity of 8 Mbps BW in the presence of an SPTCP CUBIC flow as background traffic. As we can observe, SF2 successfully utilizes that available BW. The same trend of yield is observed throughout the experiment, which demonstrates the efficacy of the proposed BA-MPCUBIC algorithm.

Table 2 shows the bottleneck detection time and detection accuracy of BA-MPCUBIC. We also calculated the detection accuracy and detection time for Ferlin’s SBD+MPCUBIC. One may argue that because BA-MPCUBIC implements the ECN_filter, which depends on the ECN mechanism, BA-MPCUBIC might not work or demonstrate a significant decrease in performance in the absence of the ECN mechanism. Therefore, we performed experiments using the same scenarios wherein we disabled the ECN mechanism and calculated the SB and NSB detection accuracy and time, as presented in Table 2. We calculated detection accuracy as follows:

(13) $$\mathrm{Accuracy}=\frac{\mu }{\Gamma }\times 100$$

where μ and Γ are the number of successful and total detections, respectively. The detection time implies the time required by an algorithm to successfully recognize an SB or NSB. For a static (non-changing) network scenario, the time is calculated from the start time of an SF to the time when it is determined that the SF is going through an SB or NSB. For a dynamically changing network scenario, it is calculated from the time a bottleneck link becomes the SB or NSB to the time when the algorithm successfully determines the changed bottleneck condition.

From Table 2 it is evident that BA-MPCUBIC shows the best detection accuracy in all scenarios. Even in the absence of an ECN mechanism, BA-MPCUBIC achieves better accuracy than the compared algorithm. Moreover, in the case of detection time, it takes a shorter time than the competitor even in the absence of an ECN mechanism. The Ferlin’s SBD+MPCUBIC SB detection scheme primarily rely only on OWD. The state-of-the-art BA-MPCUBIC implements three filters related to RTT, ECN, and packet loss. This enables the proposed BA-MPCUBIC algorithm to understand and utilize all the available parameters in a systematic manner to produce a better detection capability. Even in the absence of an ECN mechanism, the other two filters successfully keep the detection process on the right track.

## 5. Conclusions

In this work, we addressed the problem of developing an MPTCP CCA that successfully satisfies the MPTCP CCA design goals. The existing MPTCP CCAs are too fair to SPTCP flows; thus, they do not utilize the available resources to their full extent. Although CUBIC has been widely accepted as a successful SPTCP CCA, an MPTCP variant that can eliminate the existing limitations while fulfilling the design goals is still not available. We address these issues by implementing BA-MPCUBIC, a multipath CUBIC variant that implements SPTCP CUBIC for SFs going through NSBs and multipath CUBIC for SFs traveling via SBs. We also implemented an innovative state-of-the-art SB detection technique that successfully isolates the flows going through SBs from NSBs based on RTT, ECN, and packet loss.

We implemented BA-MPCUBIC in the Linux kernel and conducted extensive emulation experiments in various scenarios concerning real-world dynamic networks. We found that the proposed BA-MPCUBIC algorithm successfully satisfies the MPTCP CCA design goals by better utilizing the underlying network while ensuring fairer behavior to SPTCP flows. In addition, the proposed SB detection technique yielded the highest detection accuracy with the lowest detection time.

In the future, we plan to present a detailed experimental and mathematical analysis on fine-tuning the internal parameters of BA-MPCUBIC. We also plan to further improve BA-MPCUBIC such that it can detect SBs in a much shorter time. Moreover, we believe that the proposed SB detection method can be further extended to implement other SPTCP CCAs for MPTCP.

## Figures and Tables

**Table 1:** Performance comparison in terms of overall Agre_Bft and fairness index in Scenarios #1–5.

	BA-MPCUBIC		Ferlin’s SBD+MPCUBIC		Le’s MPCUBIC		U-MPCUBIC		LIA		OLIA		BALIA	
	*Agre_Bft*	Fairness Index	*Agre_Bft*	Fairness Index	*Agre_Bft*	Fairness Index	*Agre_Bft*	Fairness Index	*Agre_Bft*	Fairness Index	*Agre_Bft*	Fairness Index	*Agre_Bft*	Fairness Index
Scenario #1	0.37	0.97	0.19	0.97	0.04	0.96	0.42	0.98	−0.22	0.91	−0.20	0.91	0.03	0.92
Scenario #2	0.25	0.98	0.43	0.93	0.18	0.98	0.63	0.83	−0.18	0.92	−0.11	0.94	−0.13	0.92
Scenario #3	0.48	0.95	0.27	0.93	−0.18	0.93	0.73	0.83	−0.43	0.81	−0.23	0.81	−0.21	0.82
Scenario #4	0.34	0.88	0.01	0.81	−0.23	0.69	0.70	0.64	−0.47	0.69	−0.42	0.72	−0.30	0.71
Scenario #5	0.32	0.89	−0.08	0.86	−0.34	0.76	0.62	0.74	−0.54	0.76	−0.47	0.77	−0.42	0.77

**Table 2:** Performance comparison in terms of detection accuracy and detection time in Scenarios #1–5.

		BA-MPCUBIC		BA-MPCUBIC (ECN Disabled)		Ferlin’s SBD+MPCUBIC	
		Accuracy (%)	Time (ms)	Accuracy (%)	Time (ms)	Accuracy (%)	Time (ms)
Scenario #1	B1-1 (NSB)	99.3	1750	98.1	2000	96.2	2520
	B1-2 (NSB)	99.5	1750	98.2	2000	95.5	2857
Scenario #2	B1 (SB)	99.8	1500	99.2	1500	98.1	2018
Scenario #3	B1 (NSB)	98.6	2000	96.8	2000	91.2	2854
	B2 (SB)	98.3	1750	95.1	2250	89.6	3328
Scenario #4	B1 (SB/ NSB)	97.2	2500	94.5	2750	85.6	4151
	B2 (SB/NSB)	97.4	2250	94.2	2750	86.4	3922
Scenario #5	B1 (SB)	97.6	2000	95.1	3250	83.4	3899
	B2 (SB/NSB)	97.1	2250	94.2	3000	83.2	4563
	B3 (SB/NSB)	96.9	2750	93.8	3000	84.1	4321
	B4 (NSB)	97.8	1750	95.6	2750	89.1	2517
